# Two-Steps Method to Prepare Multilayer Sandwich Structure Carbon Fiber Composite with Thermal and Electrical Anisotropy and Electromagnetic Interference Shielding

**DOI:** 10.3390/ma16020680

**Published:** 2023-01-10

**Authors:** Chuanqi Zhang, Lansen Bi, Song Shi, Huanhuan Wang, Da Zhang, Yan He, Wei Li

**Affiliations:** 1 College of Electromechanical Engineering, Qingdao University of Science and Technology, Qingdao 266061, China; 2 Shandong Laboratory for Preparation and Application of High-Performance Carbon Materials, Qingdao 266061, China; 3 Shandong Collaborative Innovation Center of Intelligent Green Manufacturing Technology and Equipment, Qingdao 266061, China; 4 Department of Energy Engineering, Zhejiang University, Hangzhou 310027, China

**Keywords:** carbon nanotubes, structural composites, plasma spraying

## Abstract

Carbon fiber (CF) composites performance enhancement is a research hotspot at present. In this work, first, a sandwich structure composite, CF@(carbon nanotube/Fe_3_O_4_)/epoxy (CF@(CNT/Fe_3_O_4_)/EP), is prepared by the free arc dispersion-CFs surface spraying-rolling process method, herein, CFs in the middle layer and (CNT/Fe_3_O_4_)/EP as top and substrate layer. Then, CF@(CNT/Fe_3_O_4_)/EP (on both sides) and CFs (in the middle) are overlapped by structure design, forming a multilayer CF@(CNT/Fe_3_O_4_)/EP-CFs composite with a CFs core sheath. A small amount of CNT/Fe_3_O_4_ is consumed, (CNT/Fe_3_O_4_)/EP and CFs core sheath realize thermal and electrical anisotropy and directional enhancement, and multilayer sandwich structure makes the electromagnetic interference (EMI) shielding performance better strengthened by multiple absorption–reflection/penetration–reabsorption. From CF-0 to CF-8, CNT/Fe_3_O_4_ content only increases by 0.045 wt%, axial thermal conductivity (λ_‖_) increases from 0.59 W/(m·K) to 1.1 W/(m·K), growth rate is 86%, radial thermal conductivity (λ_⊥_) only increases by 0.05 W/(m·K), the maximum λ_‖_/λ_⊥_ is 2.9, axial electrical conductivity (σ_‖_) increases from 6.2 S/cm to 7.7 S/cm, growth rate is 24%, radial electrical conductivity (σ_⊥_) only increases by 0.7 × 10^−4^ S/cm, the total EMI shielding effectiveness (EMI SE_T_) increases by 196%, from 10.3 dB to 30.5 dB. This provides a new idea for enhancing CFs composite properties.

## 1. Introduction

With the rapid development of aerospace, transportation, energy, medical and health fields, there is an urgent need for materials with excellent thermal/electrical conductivity properties and electromagnetic interference shielding effectiveness (EMI SE) to adapt to the work in complex environments. Carbon fiber (CF) composites are widely used because of its high strength, high modulus, light weight and easy molding [1,2,3,4,5]. However, the poor magnetic property for CFs limits the further improvement of EMI SE [6]. In addition, the epoxy resin (EP), which is often used as the matrix of CF composites, has advantages of light weight, designability and easy processing [7], but its low intrinsic thermal conductivity (0.1~0.4 W/(M·K)) [8,9] and EMI SE (about 2 dB) [10] limit the performance of CF composites. Therefore, the preparation of CF composites with excellent thermal/electrical conductivity properties and EMI SE has become a research hotspot.

Adding nanofillers is one of the effective methods to prepare high performance composites [11,12,13]. Carbon nanotubes (CNTs) have excellent thermal and electrical properties [14,15] and are often used as an ideal material to enhance thermal/electrical conductivity properties of CF composites [16,17]. Moreover, because of their good dielectric loss characteristic, CNTs are a good choice for EMI shielding materials [18,19,20], and CFs have similar characteristic [21,22,23]. However, if CNTs and CFs are simply combined, although CF-CNT composites have good thermal and electrical properties, lacking magnetic property and impedance mismatch [6] will make CF-CNT composites have weak electromagnetic wave absorption and high electromagnetic reflectance, which result in secondary electromagnetic pollution [24,25]. The matching of electrical and magnetic properties is the key to obtain good shielding effect in the wide frequency range [26]. Therefore, in order to supplement the magnetic property lacking for CFs and CNTs and enhance magnetic loss, Fe_3_O_4_, Fe_2_O_3_, Fe, Ni, Co and other magnetic particles are usually introduced to cooperate with CNTs [27,28,29,30]. Main methods include in situ growth [31,32] and mechanical blending [33,34]. Among of them, Fe_3_O_4_ has a low toxicity and good biocompatibility as a more efficient shielding material. Furthermore, because of the large saturation magnetization of Fe_3_O_4_, they can provide a high value of complex permeability. Fe_3_O_4_ can exhibit the skin effect, their high resistivity allowing the electromagnetic waves to enter effectively. Therefore, CNT/Fe_3_O_4_ as a material with dual magnetic and dielectric properties could be important to achieve excellent thermal, electrical and EMI shielding effectiveness. On this basis, Li et al. [35,36] propose a free arc dispersion method, which can rapidly disperse nanomaterials and produce nanomaterials dispersion fog with good dispersion degree in the air. In addition, this method can disperse variety of nanomaterials at the same time, and the dispersed nanomaterials have a good mixing effect. This seems to well meet the need of collaborative use for the Fe_3_O_4_ and CNTs.

Another method to prepare high-performance composites is structure design to obtain composites with specific functions [37,38,39,40,41,42,43]. On the one hand, in order to meet the requirements of directional heat dissipation or electrical conductivity for composites, composites are required to have anisotropy [37,38]. On the other hand, the single-layer shielding structure is not easy to achieve high absorption loss, so sandwich structure, multilayer structure and porous structure begin to appear [39,40,41,42]. Based on the difference between the radial and axial thermoelectric properties of CFs and CNTs, if CNTs are extended along the CFs axial direction to form a CNTs network that is attached to the CFs surface, meanwhile, the Fe_3_O_4_ is mixed in the CNTs network by the free arc dispersion method, and CF composites with both thermal and electrical anisotropy and the EMI shielding property can be obtained. Furthermore, multilayer CF composites constructed by the above material can not only achieve the directional enhancement of thermal and electrical anisotropy for CF composites but also realize EMI shielding performance enhancement by multiple absorption–reflection/penetration–reabsorption when electromagnetic waves pass through each layer of CF composites.

Here, as step one, CF@(CNT/Fe_3_O_4_)/EP with sandwich structure is prepared by the free arc dispersion-CFs surface spraying-rolling process method, CFs in the middle layer, and (CNT/Fe_3_O_4_)/EP as top and substrate layer. Step two, CF@(CNT/Fe_3_O_4_)/EP (on both sides) and CFs (in the middle) are overlapped by structure design, forming multilayer CF@(CNT/Fe_3_O_4_)/EP-CFs composite with CFs core sheath. The structural morphology of CNT/Fe_3_O_4_ and CF@(CNT/Fe_3_O_4_)/EP are characterized by scanning electron microscopy (SEM), Raman spectroscopy and X-ray diffraction (XRD). The influence of multilayer sandwich structure on thermal and electrical anisotropy and EMI SE of multilayer CF@(CNT/Fe_3_O_4_)/EP-CFs composite is studied.

## 2. Materials and Experiments

### 2.1. Materials

CNTs (GT-300, length 15–30 μm, diameter 5–15 nm) were provided by Shandong Dazhan Nano Materials Co., Ltd., Binzhou, China. Fe_3_O_4_ (diameter 20 nm) was purchased from Nanjing Emperor Nano Materials Co., Ltd., Nanjing, China. CF (T700SC, diameter 7 μm) was supplied from Lianyungang Zhongfu Shenying Carbon Fiber Co., Ltd., Lianyungang, China. Epoxy resin (MF-4101H) and curing agent (ZH-520), Curing temperature T_1_ = 150 °C, T_2_ = 180 °C, curing time T_1_ = 2 h, 2 = 2 h, was obtained from Hubei Zhen Zhengfeng Advanced Materials Co., Ltd., Huanggang, China. Deionized water (DI water) was used as a dispersive working medium.

### 2.2. Experiments

Step1, free arc dispersion-CFs surface spraying-rolling process method

CNTs, Fe_3_O_4_ and DI water were mixed at a mass ratio of 1:3:10 and thoroughly stirred for 10 min, putting in the mold and applying 10 kg pressure to extrude into a cylindrical block (diameter 30 mm and height 10 mm). According to the free arc dispersion method of Li et al. [35,36], the cylindrical block was placed between the high-voltage pulse electrodes for dispersion, the voltage was 12 KV, the frequency was 10 Hz, the positive electrode used titanium grid, the negative electrode used titanium plate and CNT/Fe_3_O_4_ dispersion fog was obtained. At the same time, CNT/Fe_3_O_4_ dispersion fog passed through the spraying channel and was sprayed on continuously moving CFs surface by negative pressure airflow traction, and CFs movement speed was 0.01 m/s. The sprayed CFs moved into the heating box for heating at 100 °C to obtain CF@(CNT/Fe_3_O_4_). Finally, EP was poured on CF@(CNT/Fe_3_O_4_), after rolling, CNT/Fe_3_O_4_ was laid on the CFs surface to construct CNT/Fe_3_O_4_ network and CF@(CNT/Fe_3_O_4_)/EP was prepared. The above processes were simultaneous and continuous.

Step2, structure design

Pure CFs was placed in the middle as the core sheath, and CF@(CNT/Fe_3_O_4_)/EP was overlapped on the upper and lower sides of pure CFs, putting into the mold, and transferring to the heating box for curing, multilayer CF@(CNT/Fe_3_O_4_)/EP-CFs composite was obtained. Curing temperature T_1_ = 150 °C, T_2_ = 180 °C, curing time T_1_ = 2 h, T_2_ = 2 h. The sample size was 2 mm × 12 mm × 20 mm.

After calculation, the CFs volume fraction was 60% and the volume fraction of EP was 40% in the sample. Considering the sample performance gradient and consistency of composite size, the total number of layers for CF@(CNT/Fe_3_O_4_)/EP and pure CFs was fixed to 8. The schematic diagram of CFs overlapping method and the description of treatment for each experimental group were shown in Table 1.

### 2.3. Characterizations

Field emission scanning electron microscope SEM (SU-8010, Hitachi, Tokyo, Japan) was applied to observe the surface distribution and morphology of CFs and composites. Raman spectrometer (InVia Reflex, Renishaw, London, UK) was used to analyze the material structure of CNT and Fe_3_O_4_, and the laser wavelength was 532 nm. X-ray diffractometer XRD (MiniFlex 600, Rigaku, Tokyo, Japan) was used to characterize the atomic structure of CNT and Fe_3_O_4_, and the scanning speed was 10°/min, the range was 20–80°. Thermal constant analyzer (TPS2500S, Hot Disk, Uppsala, Sweden) was used to test the thermal conductivity of multilayer CF@(CNT/Fe_3_O_4_)/EP-CFs composite according to the standard of ISO22007-2-2015. Electrical conductivity of multilayer CF@(CNT/Fe_3_O_4_)/EP-CFs composite was measured by four probes resistance tester (RTS-8, Guangzhou Four Probes Technology, Guangzhou, China), and micro-current tester (ST2643, Suzhou Jingge, Suzhou, China) was used to test interlaminar resistivity of multilayer CF@(CNT/Fe_3_O_4_)/EP-CFs composite. Vibrating sample magnetometer VSM (7404, LakeShore, OH, USA) was employed to test the magnetization hysteresis loops of CFs, CNT/Fe_3_O_4_ and CF@(CNT/Fe_3_O_4_)/EP at room temperature. Vector network analyzer (ZNB20, Rohde & Schwarz, Munich, Germany) was employed to measure the *S*_11_, *S*_22_, *S*_12_ and *S*_21_ parameters of multilayer CF@(CNT/Fe_3_O_4_)/EP-CFs composite according to the standard of ASTM D5568-08, frequency was X-band (8.2–12.4 GHz). the total EMI SE (SE_T_), reflection EMI SE (SE_R_) and the absorption EMI SE (SE_A_) of multilayer CF@(CNT/Fe_3_O_4_)/EP-CFs composite were calculated according to the following formula [44]:

(1) $${\mathrm{SE}}_{T}=10\mathrm{lg}\left(\frac{1}{{\left|S_{12}\right|}^{2}}\right)$$

(2) $${\mathrm{SE}}_{R}=10\mathrm{lg}\left(\frac{1}{1-{\left|S_{11}\right|}^{2}}\right)$$

(3) $${\mathrm{SE}}_{A}=10\mathrm{lg}\left(\frac{1-{\left|S_{11}\right|}^{2}}{{\left|S_{12}\right|}^{2}}\right)$$

## 3. Results and Discussion

**Figure 1:**
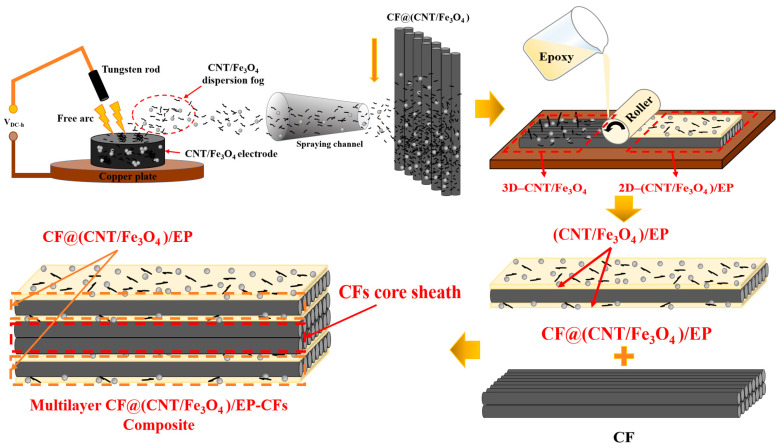
Schematic diagram of CF@(CNT/Fe_3_O_4_)/EP composite preparation process.

Figure 1 is the schematic diagram of multilayer CF@(CNT/Fe_3_O_4_)/EP-CFs composite preparation process. “Free arc dispersion” can disperse CNTs and Fe_3_O_4_ at the same time and obtain the CNT/Fe_3_O_4_ dispersion fog with well mix and dispersion degree. “CFs surface spraying” can spay CNT/Fe_3_O_4_ dispersion fog onto the CFs surface rapidly. “Rolling process” can flatten the 3D-CNT/Fe_3_O_4_ and form the 2D-(CNT/Fe_3_O_4_)/EP layer, while making CNTs has direction, which is beneficial to enhance the interlayer insulation performance, achieving thermal and electrical anisotropy of CF composites. Based on this, a sandwich structure composite (CF@(CNT/Fe_3_O_4_)/EP) with CFs in the middle layer and (CNT/Fe_3_O_4_)/EP as top and substrate layer is prepared. Furthermore, through the structure design, the CFs is placed in the middle as core sheath and CF@(CNT/Fe_3_O_4_)/EP is placed on both sides to prepare a multilayer sandwich structure CF composite (multilayer CF@(CNT/Fe_3_O_4_)/EP-CFs composite).

**Figure 2:**
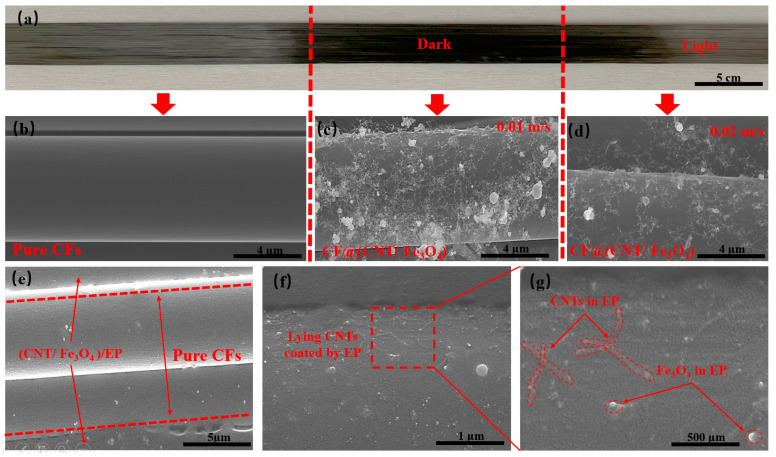
(**a**) Pure CFs at the left, CF@(CNT/Fe_3_O_4_) with high content CNT/Fe_3_O_4_ at middle, and low content CNT/Fe_3_O_4_ at right. (**b**) SEM of pure CFs. (**c**) CF@(CNT/Fe_3_O_4_) with no rolling and high content CNT/Fe_3_O_4_, and (**d**) with no rolling and low content CNT/Fe_3_O_4_. (**e**) CF@(CNT/Fe_3_O_4_)/EP with rolled treatment, CFs in the middle and CF@(CNT/Fe_3_O_4_) on both sides. (**f**,**g**) CF@(CNT/Fe_3_O_4_)/EP locally enlarged image; lying CNTs are coated by EP.

As is shown in Figure 2a, the left side is pure CFs, and the middle and right side are the CFs sprayed with CNT/Fe_3_O_4_ dispersion fog. It can be clearly seen that pure CFs has a light color and luster, but the CFs sprayed with CNT/Fe_3_O_4_ dispersion fog appears darker color. This is because the adsorption of CNTs and Fe_3_O_4_ on the CFs surface and changes the diffuse reflection of CFs surface. Figure 2b shows that pure CFs has a smooth surface without any substance. Figure 2c,d show the attachment of CNT/Fe_3_O_4_ when CFs moving speed is 0.01 m/s and 0.02 m/s, respectively. The faster CFs moving speed, the less CNT/Fe_3_O_4_ is deposited, and the lighter color is appeared on the macroscopic (Figure 2a, right). At the same time, CNTs and Fe_3_O_4_ have high dispersion degree and without obvious agglomeration, CNTs is connected to each other and extend to the radial and axial directions of CFs, presenting a 3D distribution, Fe_3_O_4_ is interspersed in the CNTs network, and adsorbed on the CFs surface. On the one hand, CNTs and Fe_3_O_4_ are coated by the size agent on the CFs surface, which establishes the physical association between CFs and CNT/Fe_3_O_4_ [45]. On the other hand, this may be related to the high specific surface area of CFs [46]. Figure 2e shows the sandwich structure CF@(CNT/Fe_3_O_4_)/EP composite obtained after rolling, with CFs in the middle and the thickness of (CNT/Fe_3_O_4_)/EP distributed on both sides is about 2 µm, which is uniformly attached to the CFs surface. It can be clearly seen in Figure 2f,g that CNTs and Fe_3_O_4_ are coated in EP, in which CNTs is attached to the CFs surface and distribute in the axial direction of CFs only, and Fe_3_O_4_ is interspersed in CNTs network with uniform distribution. This morphology is obviously different from Figure 2c,d; this indicates that the effect of rolling makes CNT/Fe_3_O_4_ change from 3D to 2D planar structure, which is conducive to maintaining the insulation between CFs layers.

**Figure 3:**
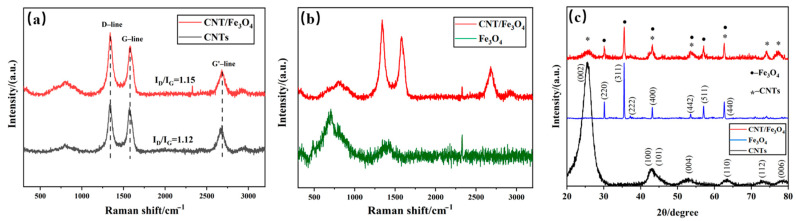
Raman spectral comparison of CNT/Fe_3_O_4_ and CNTs (**a**), and CNT/Fe_3_O_4_ and Fe_3_O_4_ (**b**), XRD of Fe_3_O_4_, CNTs and CNT/Fe_3_O_4_ (**c**).

Considering that CNTs and Fe_3_O_4_ may change their properties under the action of free arc, Fe_3_O_4_ may be converted into Fe_2_O_3_ at high temperature [47]. The Raman of CNT/Fe_3_O_4_ dispersion fog obtained using the free arc dispersion method is compared with pure CNTs (Figure 3a). CNT/Fe_3_O_4_ dispersion fog has characteristic peaks at 1341 cm^−1^ (D-line) and 1578 cm^−1^ (G-line), which are the characteristic peaks of carbonaceous compounds [48,49]; this parameter complies with the CNT standard spectrum. In addition, the I_D_/I_G_ values of CNTs/Fe_3_O_4_ and CNTs are 1.15 and 1.12, respectively; this indicates that the graphitization degree of CNTs is not affected by the free arc. Figure 3b shows the comparison of CNT/Fe_3_O_4_ dispersion fog Raman image and Fe_3_O_4_ standard spectrum, and the result is also consistent [50,51]. It demonstrated that the structure of CNTs and Fe_3_O_4_ do not change, and CNT/Fe_3_O_4_ dispersion fog has a higher purity, only containing CNTs and Fe_3_O_4_; there are no other substances. XRD image (Figure 3c) shows that the diffraction peaks of CNTs and Fe_3_O_4_ are in good agreement with CNT/Fe_3_O_4_ dispersion fog, respectively; there are corresponding diffraction peaks at particular diffraction angles [52,53]. The above characterizations indicate that CNTs and Fe_3_O_4_ maintain good material structure during free arc dispersion and spraying.

**Figure 4:**
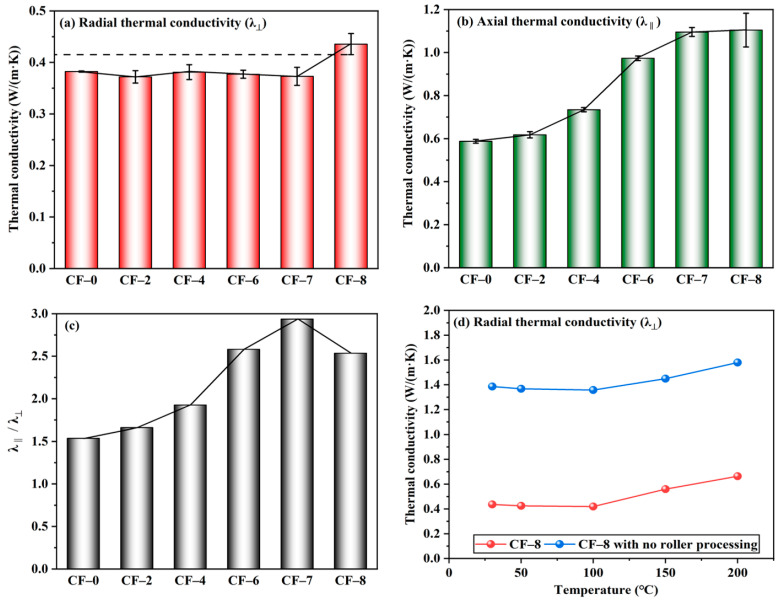
Radial thermal conductivity λ_⊥_(**a**), axial thermal conductivity λ_‖_ (**b**), and λ_‖_/λ_⊥_(**c**) of multilayer CF@(CNT/Fe_3_O_4_)/EP-CFs composite, λ_⊥_ comparison of roll treatment (**d**).

Radial thermal conductivity (λ_⊥_) of the multilayer CF@(CNT/Fe_3_O_4_)/EP-CFs composite is shown in Figure 4a. The λ_⊥_ of CF-0 is 0.38 W/(m·K), and the λ_⊥_ of CF-2 to CF-7 remains stable at about 0.38 W/(m·K) with the increase of CNT/Fe_3_O_4_. This is because CNT/Fe_3_O_4_ changes from 3D to 2D plane due to the rolling treatment. CNTs with excellent thermal conductivity (about 3000 W/(m·K)) [54] are attached to the CF surface and covered by EP [8] with high insulation, forming (CNT/Fe_3_O_4_)/EP. It makes cross-plane heat conduction in the multilayer CF@(CNT/Fe_3_O_4_)/EP-CFs composite not easy. In addition, due to the presence of contact thermal resistance between CFs [55], the CFs core sheath in CFs 2 to CFs 7 forms radial thermal insulation layer. Both (CNT/Fe_3_O_4_)/EP and CFs core sheath form the multilayer thermal insulation system. λ_⊥_ of CF-8 increases slightly. From CF-7 to CF-8, λ_⊥_ increases from 0.38 W/(m·K) to 0.44 W/(m·K). The main reason is that CF-8 does not contain a CFs core sheath; therefore, the radial thermal insulation layer is lost, but because of the (CNT/Fe_3_O_4_)/EP, the increase of λ_⊥_ is not significant.

Axial thermal conductivity (λ_‖_) of the multilayer CF@(CNT/Fe_3_O_4_)/EP-CFs composite is shown in Figure 4b. Different from λ_⊥_, with the increase of CNT/Fe_3_O_4_ content, λ_‖_ increases. When the amount of CNT/Fe_3_O_4_ is from 0 to 0.56 mg/cm^3^ (CF-0 to CF-7), λ_‖_ increases from 0.59 W/(m·K) to 1.1 W/(m·K), and the growth rate is 86%. The reason is that CFs has excellent thermal conductivity [56], and the axial heat conduction is not affected by EP and CFs core sheath. In addition, CNTs in the (CNT/Fe_3_O_4_)/EP forms a good thermal conductivity network [57]; the higher CNTs content, the more abundant CNTs network, and the higher heat conduction efficiency. When the amount of CNT/Fe_3_O_4_ is changed from 0.56 to 0.64 mg/cm^3^ (CF-7 to CF-8), λ_‖_ hardly changes, but the instability (standard deviation) increases. The main reason is that CF-8 does not have CFs core sheath, the barrier of radial heat conduction is greatly reduced, so the heat conduction has a component in radial. Thus, the increase in axial thermal conductivity is limited and the heat transfer randomness is increased.

The difference between λ_‖_ and λ_⊥_ indicates that the CFs core sheath and (CNT/Fe_3_O_4_)/EP have influence on the thermal anisotropy of multilayer CF@(CNT/Fe_3_O_4_)/EP-CFs composite. Figure 4c directly represents the difference between λ_‖_ and λ_⊥_of multilayer CF@(CNT/Fe_3_O_4_)/EP-CFs composite, the larger value of λ_‖_/λ_⊥_, the more significant thermal anisotropy. From CF-0 to CF-7, λ_‖_/λ_⊥_ gradually increases, while CF-7 to CF-8 starts to decrease. Obviously, the λ_‖_/λ_⊥_ of CF-7 is higher than CF-8; this is attributed to the CFs core sheath in CF-7. On the one hand, the CFs core sheath stabilizes λ_⊥_; on the other hand, the CFs core sheath eliminates the radial component of heat conduction to ensure λ_‖_ promotion. For CF-8, the large λ_⊥_ and the similar λ_‖_ make its thermal anisotropy insignificant compared to CF-7. Figure 4d shows that within the 30–200 °C, the λ_⊥_ of CF-8 with rolling treatment (about 0.4 W/(m·K)) is lower than no rolling treatment (about 1.4 W/(m·K)). This shows from the performance point of view that the (CNT/Fe_3_O_4_)/EP formed by rolling can effectively reduce the heat transfer between layers. Combined with the difference between λ_‖_ and λ_⊥_, the main reason is that CNTs have high axial thermal conductivity [58], and rolling makes CNTs attach to the CFs surface and extend along the axial direction of CFs. At this time, heat can be transferred along the CFs axial direction; however, due to the coverage of EP and the direction of CNTs, radial heat transfer is difficult.

**Figure 5:**
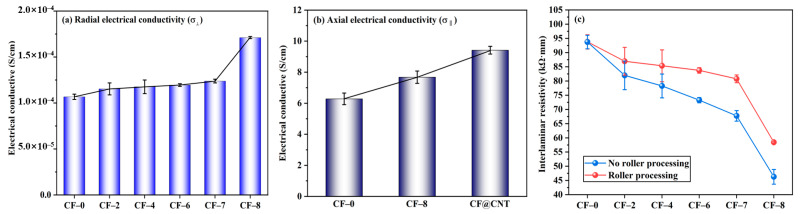
Radial electrical conductivity σ_⊥_ of multilayer CF@(CNT/Fe_3_O_4_)/EP-CFs composite (**a**), axial electrical conductivity σ_‖_comparison of CF, CF@ (CNT/Fe_3_O_4_) and CF@CNT composite (**b**), interlaminar resistivity comparison of roll treatment (**c**).

Radial electrical conductivity (σ_⊥_) of multilayer CF@(CNT/Fe_3_O_4_)/EP-CFs composite is shown in Figure 5a. The σ_⊥_ of CF-0 to CF-7 is generally stable, maintaining at 1.1 × 10^−4^ (S/cm), while the σ_⊥_ of CF-8 is increased to 1.7 × 10^−4^ (S/cm), showing a slight improvement. The main reason is that CNTs has low resistance/high electrical conductivity (10^5^–10^7^ S/m) [59,60], and adding CNTs to the composite can improve electrical conductivity. Similar to the thermal conductivity, due to the insulation effect of (CNT/Fe_3_O_4_)/EP and CFs core sheath, the cross-plane electrical conduction in multilayer CF@(CNT/Fe_3_O_4_)/EP-CFs composite is difficult to carry out. Because CF-8 does not have CFs core sheath and CNTs enhance the electrical conductivity of EP [61], so the σ_⊥_ of CF-8 obtains some improvement.

As shown in Figure 5b, axial electrical conductivity (σ_‖_) is higher than σ_⊥_, the σ_‖_ of CF-0, CF-8 and CF@CNTs (the content of CNTs is 0.64 mg/cm^3^) are 6.2 S/m, 7.7 S/m and 9.4 S/m, respectively, showing increase trend. The main reason is that σ_‖_ is not restricted by (CNT/Fe_3_O_4_)/EP, CFs core sheath and interlamination contact resistance, and CFs have high axial electrical conductivity (about 670 S/cm) [62]. The σ_‖_ of CF-8 is higher than CF-0 because CNTs is contained in the filler, and the CNTs direction is along the CFs axial direction, which helps to improve the axial electrical conductivity of EP and composite. In addition, although the same mass of CNTs (0.64 mg/cm^3^) is added in CF@CNT composite, the σ_‖_ of CF@CNT is higher than CF-8. This is attributed to the fact that Fe_3_O_4_ has poor electrical conductivity [63], CF-8 contains 0.48 mg/cm^3^ Fe_3_O_4_ and CNTs content is much lower than CF@CNT, which makes low electrical conductivity for CF-8.

Figure 5c shows that the interlaminar resistivity of multilayer CF@(CNT/Fe_3_O_4_)/EP-CFs composite with rolling is generally higher than no rolling. The main reason is that CNTs no rolling may penetrate EP, thus connecting adjacent CFs, forming CFs-CNTs-CFs interlayer electric conduction pathway, which reduces the macroscopic resistivity and influences the interlamination insulation performance of the composite.

**Figure 6:**
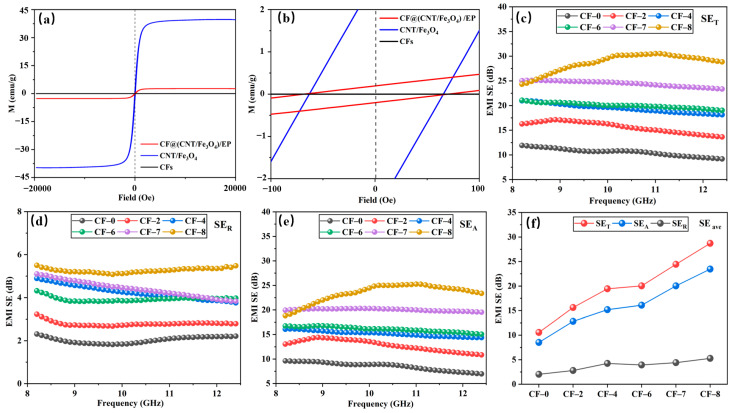
Magnetic hysteresis loops at room temperature (**a**,**b**), EMI SE_T_ (**c**), EMI SE_R_ (**d**), EMI SE_A_ (**e**) and EMI SE_ave_ (**f**) at 8.2 to 12.4 GHz.

The magnetic property of CFs, CNT/Fe_3_O_4_ powder and CF@(CNT/Fe_3_O_4_)/EP are tested, and the results are shown in Figure 6a. CFs has no magnetic, and the saturation magnetization (Ms) of CNT/Fe_3_O_4_ powder is 40 emu/g, when combined with CFs and EP, the Ms decreases to 2.6 emu/g, which is mainly attributed to CNT/Fe_3_O_4_ is coated [64]. It can be seen from local magnification (Figure 6b) that the coercivity (Hc) of CNT/Fe_3_O_4_ powder and CF@(CNT/Fe_3_O_4_)/EP are 66.6Oe and 64.6Oe, respectively. The similar Hc values indicate that free arc has no effect on the antidemagnetization ability of CNT/Fe_3_O_4_.

The SE_T_, SE_R_ and SE_A_ of CF-0 to CF-8 are shown in Figure 6c–e. The higher CNT/Fe_3_O_4_ content, the higher SE_T_, SE_R_ and SE_A_ value, and the SE_A_ value is greater than SE_R_. In CF@(CNT/Fe_3_O_4_)/EP, electromagnetic wave interacts with Fe_3_O_4_ first when passing through (CNT/Fe_3_O_4_)/EP, part of the electromagnetic wave is absorbed due to hysteresis loss and natural resonance, and the rest will reach the CFs surface. Here, a part of electromagnetic wave is reflected back to (CNT/Fe_3_O_4_)/EP due to impedance mismatch, and the remaining part will pass through CFs to (CNT/Fe_3_O_4_)/EP on the other side [55]. CF@(CNT/Fe_3_O_4_)/EP with sandwich structure attenuates electromagnetic wave by multiple absorption, reflection and scattering processes and improves its EMI shielding performance [65,66,67]. Due to multilayer CF@(CNT/Fe_3_O_4_)/EP-CFs composite having more than one layer of CF@(CNT/Fe_3_O_4_)/EP, it provides more opportunities for electromagnetic wave propagation, so the above attenuation process of absorption–reflection/penetration–reabsorption for electromagnetic wave will be repeated many times and strengthens EMI shielding performance. In this process, since absorption is the main attenuation mode of electromagnetic wave, so the value of SE_A_ is greater than SE_R_. In addition, as the electromagnetic wave is absorbed, the heat (converted by the electromagnetic wave) generated by the electrical loss and magnetic loss accumulates inside the composite, which will cause the composite temperature increase.

Figure 6f shows the average values (SE_ave_) of SE_T_, SE_R_ and SE_A_ in X-band (8.2–12.4 GHz). When the amount of CNT/Fe_3_O_4_ is 0 (CF-0), the SE_ave_ of SE_T_, SE_R_ and SE_A_ are 10.56 dB, 2.03 dB and 8.53 dB, respectively. When the addition of CNT/Fe_3_O_4_ is increased to 0.64 mg/cm^3^ (CF-8), compared with CF-0, the SE_ave_ of SE_T_, SE_R_ and SE_A_ are increased by 172%, 159% and 175% respectively. The reason is that CF-8 contains Fe_3_O_4_ while CF-0 does not, so the magnetic loss and dielectric loss for Fe_3_O_4_ are missing, which greatly reduces the electromagnetic wave absorption effect and leads to relatively poor EMI shielding performance [68,69].

Table 2 summarizes the λ, σ and EMI SE for some related polymer composites; it is observed that multilayer CF@(CNT/Fe_3_O_4_)/EP-CFs composite prepared by this work has good performances.

## 4. Conclusions

In this work, the multilayer CF@(CNT/Fe_3_O_4_)/EP-CFs composite is obtained by free arc dispersion-CFs surface spraying-rolling process method and structural design. Under circumstance of the content for CNT/Fe_3_O_4_ is very small, the (CNT/Fe_3_O_4_)/EP and CFs core sheath achieve thermal and electrical anisotropy and directional enhancement for multilayer CF@(CNT/Fe_3_O_4_)/EP-CFs composite, multilayer sandwich structure makes the EMI shielding performance better strengthened by multiple absorption–reflection/penetration–reabsorption of electromagnetic wave. From CF-0 to CF-8, the content of CNT/Fe_3_O_4_ only increases by 0.045 wt%, λ_‖_ increases from 0.59 W/(m·K) to 1.1 W/(m·K), the growth rate is 86%, λ_⊥_ only increases by 0.05 W/(m·K), and the maximum λ_‖_/λ_⊥_ is 2.9, σ_‖_ increases from 6.2 S/cm to 7.7 S/cm, growth rate is 24%, σ_⊥_ only increases by 0.7 × 10^−4^ S/cm and EMI SE_T_ increases by 196%, from 10.3 dB to 30.5 dB. This provides a new idea for enhancing CFs composite properties.

## Figures and Tables

**Table 1:** Description of each experimental group treatment.

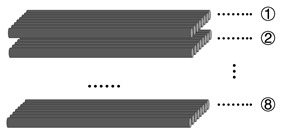	**Experiment Group**	**Spraying and Roll Treatment**	**Filler**	**The Amount of CNT + Fe_3_O_4_** **(mg/cm^3^)**	**The Content of (CNT/Fe_3_O_4_)**
	CF-0	None	None	0	0
	CF-2	①⑧	CNT and Fe_3_O_4_	0.04 + 0.12 = 0.16	0.011 wt%
	CF-4	①②⑦⑧		0.08 + 0.24 = 0.32	0.023 wt%
	CF-6	①②③⑥⑦⑧		0.12 + 0.36 = 0.48	0.034 wt%
	CF-7	①②③⑤⑥⑦⑧		0.14 + 0.42 = 0.56	0.040 wt%
	CF-8	①②③④⑤⑥⑦⑧		0.16 + 0.48 = 0.64	0.045 wt%

**Table 2:** Comparison of the λ, σ and EMI SE for some related composites.

Sample	Loading	λ (W/(m·K))	σ (S/cm)	Specific EMI SE (dB)	Thickness of Sample (mm)	Frequency Range (GHz)	Ref.
Fe_3_O_4_/CFs/Cement	0.4 wt% CF + 5wt% Fe_3_O_4_	-	-	29.8	7	8.2–12.4	[70]
CF@Fe_3_O_4_/EP	20 wt% CF@Fe_3_O_4_	-	-	22.7	2	8.2–12.4	[71]
PANI@nano-Fe_3_O_4_@CFs	5 wt% of absorbing segments	-	-	29	3	8.2–18	[72]
Gt-MWCNT/SiC/ HDPE	23.1 vol% Gt-MWCNT + 11.3 vol% SiC	-	-	14	2	8–12	[73]
RGO@GF/EP	40 wt% RGO-GF	-	-	21.3	10	8.2–12.4	[74]
PVDF@MWCNT/ BN	5 wt% MWCNT + 40 wt% BN	-	-	4.34	2	8–12	[65]
Ni@MWCNTs/HDPE	3 wt% Ni@MWCNTs	-	-	12	3	0.5–1.5	[75]
CNTs-CFs/PF	25 wt% CNTs and 40 wt% PF resin	0.636	-	-	-	-	[76]
Dry fabric/CNT mat	1.06 wt% CNT	1.386	-	-	-	-	[17]
CF + CNT	60 wt% CF and 40 wt% resin CF speed 0.01 m/s	-	1.4	-	-	-	[45]
CF@(CNT/Fe_3_O_4_)/EP-CFs	0.045 wt% CNT/Fe_3_O_4_	1.1	7.7	30.5	2	8.2–12.4	This work

## Data Availability

Not applicable.
